# Childhood abuse and borderline personality disorder features in Chinese undergraduates: the role of self-esteem and resilience

**DOI:** 10.1186/s12888-021-03332-w

**Published:** 2021-07-01

**Authors:** Guo-Die Xie, Jun-Jie Chang, Meng-Yuan Yuan, Geng-Fu Wang, Yang He, Shan-Shan Chen, Pu-Yu Su

**Affiliations:** 1 Department of Maternal, Child and Adolescent Health, School of Public Health, Anhui Medical University, No.81 Meishan Road, Hefei, 230032 Anhui China; 2 Anhui Provincial Center for Disease Control and Prevention, No.12560 Fanhua Avenue, Hefei, 230601 Anhui China; 3 Key Laboratory of Population Health Across Life Cycle (Anhui Medical University), Ministry of Education of the People’s Republic of China, No 81 Meishan Road, Hefei, 230032 Anhui China; 4 Anhui Provincial Key Laboratory of Population Health and Aristogenics, No 81 Meishan Road, Hefei, 230032 Anhui China

**Keywords:** Childhood abuse, Self-esteem, Resilience, Borderline personality disorder, Mediation effects, Structural equation model

## Abstract

**Background:**

Although childhood abuse is considered to be related to borderline personality disorder (BPD), few studies have elaborated on the mediating role of self-esteem and resilience in it. Thus, the present study aimed to explore the potential mediating role of resilience and self-esteem between childhood abuse and BPD.

**Methods:**

This cross-sectional study was conducted with 4034 college students in Anhui Province, China. Participants were asked to complete Chinese versions of the following instruments: Childhood Trauma Questionnaire–Short Form (CTQ-SF), Mclean Screening Instrument for Borderline Personality Disorder (MSI-BPD), Connor-Davidson Resilience Scale (CD-RISC), and Rosenberg Self-Esteem Scale (RSES). Structural equation modeling (SEM) was used to test the mediation effects.

**Results:**

Resilience and self-esteem were found to be mediators of all three types of childhood abuse (emotional abuse, physical abuse and sexual abuse) when the types were examined separately; however, when all three types of childhood abuse were entered into the model simultaneously, neither the indirect effects nor direct effects of physical abuse or sexual abuse were found to be significant, only the association between emotional abuse and BPD features was partially mediated by resilience and self-esteem.

**Conclusions:**

Self-esteem and resilience mediate the links between childhood abuse and BPD features, and emotional abuse is uniquely associated with BPD features.

**Supplementary Information:**

The online version contains supplementary material available at 10.1186/s12888-021-03332-w.

## Background

Borderline personality disorder (BPD) is a serious psychiatric disorder common among undergraduates, which is characterized by instability in emotion regulation, impulse control, interpersonal relationships, and self-image [1]. It is a significant contributor to self-harm and even suicide that results in a serious public health burden [2, 3]. BPD is highly prevalent among undergraduates. A recent meta-analysis including 40 studies showed that the prevalence of BPD in college samples ranged from 0.5 to 32.1% worldwide [4]. Another study showed that 15% of undergraduates screened positive for BPD in China [5].

### Childhood abuse and BPD features

A multifactorial model suggested that the development of BPD, to a large extent, is an end product of childhood traumatic experiences such as emotional abuse, physical abuse and sexual abuse [6]. Additionally, researchers have found childhood abuse to be an important predictor of BPD in adolescence and adulthood [7–9]. Individuals who experienced childhood abuse tended to report higher scores for BPD features than those without that [9].

However, examining the effects of a specific subtype of childhood abuse on BPD features without controlling for the overlap between childhood abuse subtypes does not yield accurate results because most abused children have been exposed to multiple forms of abuse, which exhibit a high rate of co-occurrence [10, 11]. Considering that previous studies have confirmed that among the different forms of childhood abuse, only emotional abuse was a unique predictor of BPD features [12–14], we hypothesized that only emotional abuse would be uniquely associated with BPD features when other forms of abuse were controlled (Hypothesis 1).

Furthermore, the potential mechanisms of how childhood abuse influences BPD features are unclear. We attempted to explore the potential mediators between childhood abuse and BPD features, which may provide some meaningful guidance for school staff and mental health professionals to prevent the development of BPD features. A growing number of studies have suggested that both resilience and self-esteem were significantly associated with childhood abuse and BPD features [15–31]. In light of the mediating role of self-esteem and resilience between childhood abuse and mental health problems [32–34], it was predicted that self-esteem and resilience might play a significant mediating role in the relationship between childhood abuse and BPD features.

### Self-esteem and resilience as mediators

As suggested by the ecological systems theory, microsystem (e.g. family environment) was the most proximal factor of individual development [15]. Childhood abuse as a hostile environment for child that may threaten the ability to cope with risk or adversity and positive self-evaluations [16]. Extant research has demonstrated a strong negative relationship between childhood abuse and self-esteem [17–19], childhood abuse and resilience [20, 21]; that is, the more childhood abuse an individual experienced, the lower his or her self-esteem and resilience. As an overall view of or feeling towards oneself as worthy or unworthy [22], self-esteem is beneficial for the development of human behavior, motivation, cognition, and emotion [23]. Sandler emphasizes that positive self-evaluation has a positive effect, whereas negative self-evaluation leads to a negative effect [16]. Furthermore, there were evidence suggesting that self-esteem may play a protective role against the development of BPD features [24–26].

Resilience, which refers to a dynamic system for the maintenance of positive adaptation in the face of trauma or adversity [27], may be another promising mediator of this relationship. High resilience has been proven to be linked to better health outcomes in the face of trauma or adversity, while low resilience has been found to lead to adverse consequences on an individual’s mental health [28, 29]. The emotional flexibility theory of resilience also suggests that resilient people can flexibly change their affective and physiological responses to match the demands of frequently changing environmental circumstances [30]; thus, high resilient people could successfully cope with adversity or risk and are less likely to have BPD features [31].

### Resilience and self-esteem

Despite resilience and self-esteem are found to be closely related [35], there are still controversies about their relationship in the literature. Some studies found that resilience could exert a positive impact on the development of self-esteem, potentially through positive affect [36, 37]; Other studies, however, found that the levels of self-esteem could predict resilience [38, 39]. Based on the preceding rationale and previous findings, we hypothesized that resilience and self-esteem mediated the relationship between childhood abuse and BPD features (Hypothesis 2). Considering the uncertainty of the relationship between self-esteem and resilience, we establish three hypothesis models (Model A, Model B, Model C). Model A: two simple mediators (resilience and self-esteem) and one three-path mediator (resilience to self-esteem). Model B: two simple mediators (resilience and self-esteem) and one three-path mediator (self-esteem to resilience). Model C: two simple mediators (resilience and self-esteem).

The objectives of this study were to develop a better understanding of the mediating role of resilience and self-esteem in the development of BPD in the context of different types of childhood abuse in Chinese undergraduates. Understanding the role of self-esteem and resilience in the association between childhood abuse and BPD features may provide school staff and mental health professionals with a number of meaningful directions for preventing the development of BPD features.

## Methods

### Participants and procedures

A multistage stratification method was used to select participants. First, we randomly selected four universities from Anhui Province of China. Then, we randomly selected 2–5 classes for each grade in four schools, and excluded undergraduates under the age of 18. In total, 4287 undergraduates were invited to complete the questionnaires anonymously; of these undergraduates, 173 undergraduates refused to participate in this survey, and 54 undergraduates were absent. After removing 26 undergraduates who accidentally missed one or more tables of the questionnaire, 4034 undergraduates completed the questionnaire effectively (response rate: 94.1%). The ethics committee of Anhui Medical University approved the study (No. 20180083). We obtained written informed consent from all participants after providing them with a complete and extensive description of the study.

### Measurements

#### The childhood trauma questionnaire–short form (CTQ-SF)

The Chinese version of the CTQ-SF [40], a self-rating instrument with 28 items, was confirmed to assess traumatic experiences before the age of 16 among Chinese population reliably and validly. The frequency with which each event occurred is rated on a 5-point scale from 1 (never) to 5 (always), with higher scores indicating a higher rate of occurrence of abuse. The questionnaire is composed of five subscales: emotional abuse, physical abuse, sexual abuse, emotional neglect and physical neglect. As the focus of our study was childhood abuse, the emotional neglect and physical neglect subscales were omitted from the analyses. In this sample, the three subscales demonstrated good internal reliability (emotional abuse: α = 0.81; physical abuse: α = 0.90; sexual abuse: α = 0.95).

#### The Connor-Davidson resilience scale (CD-RISC)

The Chinese version of the 25-item CD-RISC has been confirmed to be a reliable and valid measurement in assessing resilience among Chinese adolescents [41]. Wu et al. ‘s four-factor model suggested that CD-RISC can be conceptually divided into four major domains: (1) tolerance for stress and goal orientation, (2) adaptability and acceptance of change, (3) optimism and sense of security, (4) trust in one’s instinct [42]. Items are scored on a 5-point scale (0 = not at all and 4 = true nearly all of the time). The total score ranges from 0 to 100, and higher summed scores reflect higher resilience. In this study, the Cronbach’s α coefficient of the CD-RISC was 0.97.

#### The Rosenberg self-esteem scale (RSES)

Participants completed the RSES [43], a self-report measure of self-esteem including 10 items (positively worded items and negatively worded items). Each item is rated on a 4-point scale ranging from 1 (strongly agree) to 4 (strongly disagree). Positively worded items are reverse scored; thus, higher total scores indicate a higher level of self-esteem. The Chinese version of the RSES was demonstrated to be validated among the Chinese population [44]. The Cronbach’s α coefficient of the RSES was 0.84, which indicated that it had good internal consistency in this sample.

#### Mclean screening instrument for borderline personality disorder (MSI-BPD)

BPD feature scores were measured using the MSI-BPD [45], which has been confirmed to have good validity and high internal consistency for Chinese adolescents [46]. According to Leung et al., MSI-BPD can be conceptually divided into three major domains: affect dysregulation, impulsivity, self and interpersonal disturbances [46]. The MSI-BPD comprises 10 items, each scored on a 2-point scale, with higher scores demonstrating higher severity of BPD features. In the current sample, high internal consistency of the measure was found (α = 0.84).

### Statistical analysis

The data analyses were performed using SPSS 21.0 and AMOS 24.0 statistical software. Spearman’s correlation analysis was used to evaluate the correlations between variables. Then, structural equation modeling (SEM) was carried out to analyze the mediation effects. Based on the recommendations of Hooper et al. [47], a model was considered to have an acceptable fit if the comparative fit index (CFI) and Tucker-Lewis index (TLI) were 0.95 or above, the root mean square error of approximation (RMSEA) was less than 0.08, and the χ^2^/degrees of freedom (χ^2^/df) was less than 5.00.

## Results

### Sociodemographic characteristics

A total of 4034 students participated in the study, including 1070 (26.5%) freshmen, 1048 (26.0%) sophomores, 936 (23.2%) juniors, 980 (24.3%) seniors. The participants were aged between 18 and 23 years [mean = 20.38, standard deviation (SD) = 1.35)], and 1692 (41.9%) were females (see Table 1).

**Table 1:** Sociodemographic characteristics (*N* = 4034)

Characteristics		n	%
Age (years)	Min–max	18–23	
	Mean (SD)	20.38 (1.35)	
Gender	Male	2342	58.1
	Female	1692	41.9
Area of family residence	Urban	1567	38.8
	Rural	2467	61.2
Academic disciplines	Engineering	993	24.6
	Science	961	23.8
	Agriculture	1046	25.9
	Medicine	1034	25.6
Grade	Freshmen	1070	26.5
	Sophomores	1048	26.0
	Juniors	936	23.2
	Seniors	980	24.3

### The correlated correlations between childhood abuse, self-esteem, resilience and BPD features

As shown in Table 2, BPD features were positively correlated with the three types of childhood abuse and negatively associated with self-esteem and resilience, while the three types of childhood abuse were negatively correlated with resilience and self-esteem (*P* < 0.001).

**Table 2:** Spearman correlations between all variables (*N* = 4034)

	1	2	3	4	5	6
1 emotional abuse	–					
2 physical abuse	0.47^**^	–				
3 sexual abuse	0.40^**^	0.47^**^	–			
4 resilience	−0.19^**^	− 0.16^**^	− 0.13^**^	–		
5 self-esteem	−0.21^**^	− 0.16^**^	− 0.14^**^	0.53^**^	–	
6 BPD features	0.28^**^	0.19^**^	0.14^**^	−0.31^**^	−0.28^**^	–

^**^P<0.001, ^*^P<0.05

### The mediating effects of resilience and self-esteem in the relationship between childhood abuse and BPD features

**Fig. 1:**
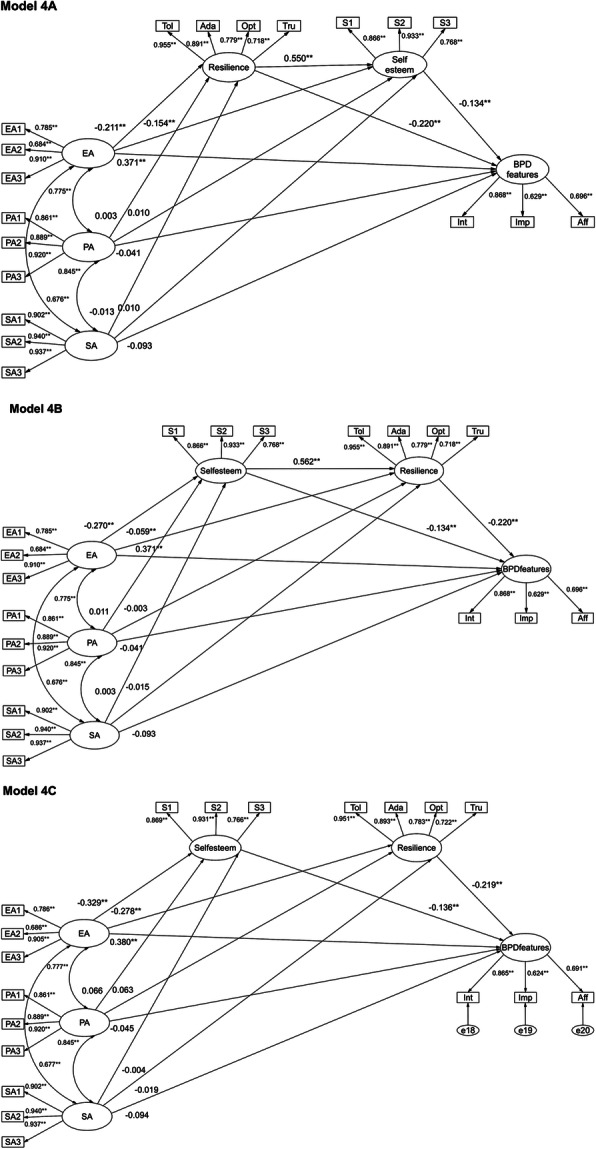
The mediating effects of resilience and self-esteem when three types of childhood abuse were examined simultaneously (Model 4). Note. This figure depicts standardized regression weights. The first model is (4a), the second model is (4b) and the third model is (4c). EA emotional abuse, PA physical abuse, SA sexual abuse. Model fit indices for Model 4A and 4B: CFI = 0.997, TLI = 0.997, RMSEA = 0.017, χ^2^ = 301.358, df = 137, χ^2^/df = 2.200; Model fit indices for Model 4C: CFI = 0.997, TLI = 0.997, RMSEA = 0.017, χ^2^ = 302.809, df = 138, χ^2^/df = 2.194. ^**^P < 0.001, ^*^P < 0.05

Each type of childhood abuse was entered into Model 1, Model 2, and Model 3 separately, and then all types of childhood abuse were simultaneously entered into one comprehensive model (Model 4). All variables were latent variables in our models. Using factorial algorithm to create separate item parcels for variables of emotional abuse, physical abuse, sexual abuse, and self-esteem [48]. Affect dysregulation, impulsivity, self and interpersonal disturbances were indicators of the latent variable of BPD features. Tolerance for stress and goal orientation, adaptability and acceptance of change, trust in one’s instinct, optimism and sense of security were indicators of the latent variable of resilience (see Fig. 1). There was evidence that the data departed significantly from multivariate normality (Model 1 to Model 4, Mardia’s coefficients were 210.163, 319.993, 623.714, 944.968) [49]; thus, these models were estimated with the maximum likelihood estimation, and fit statistics were corrected by using the Bollen-Stine bootstrapping procedure. Indirect effects and direct effects were tested by using bias-corrected bootstrapping procedures (5000 bootstrap samples and 95% confidence interval [CI]) [50, 51]. The indirect effect or direct effect was considered to be statistically significant if the bias-corrected 95% CI did not include zero [52]. The SEM results showed that all of the models had good fits (see Fig. 1, Additional files 1, 2 and 3).

When childhood emotional abuse, physical abuse or sexual abuse was considered individually (see Additional files 1, 2 and 3 for an illustration of Model 1, Model 2 and Model 3), the indirect effects and direct effects and their associated 95% CIs are shown in Additional files 4, 5 and 6. All the indirect effects and direct effects were significant, indicating that resilience and self-esteem were mediators between these three types of childhood abuse and BPD features.

When these three types of abuse were considered simultaneously (see Fig. 1 for an illustration of Model 4), the indirect effects and direct effects and their associated 95% CIs are shown in Table 3. Model 4A, Model 4B and Model 4C all showed that only the direct and indirect effects of emotional abuse on BPD features were significant (see Fig. 1 and Table 3). Consequently, when other forms of childhood abuse were controlled for, the indirect effects and direct effects of both physical abuse and sexual abuse were not found to be significant, and the association between emotional abuse and BPD features was partly mediated via resilience and self-esteem.

**Table 3:** Indirect and direct effects of childhood abuse on BPD features – three types of childhood abuse examined simultaneously (Model 4)

Model pathway	Estimate	SE	lower	upper
Model 4A - Three types of childhood abuse, two simple mediators (resilience and self-esteem) and one three-path mediator (resilience to self-esteem)				
EA → resilience → BPD features	0.046^**^	0.009	0.031	0.064
EA → self-esteem → BPD features	0.021^**^	0.005	0.013	0.032
EA → resilience → self-esteem → BPD features	0.016^**^	0.004	0.009	0.024
EA → BPD features	0.371^**^	0.044	0.289	0.458
PA → resilience → BPD features	−0.001	0.009	−0.019	0.018
PA → self-esteem → BPD features	−0.001	0.006	−0.012	0.010
PA → resilience → self-esteem → BPD features	0.000	0.003	−0.007	0.006
PA → BPD features	−0.041	0.057	−0.152	0.071
SA → resilience → BPD features	0.003	0.009	−0.014	0.019
SA → self-esteem → BPD features	−0.001	0.005	−0.012	0.008
SA → resilience → self-esteem → BPD features	0.001	0.003	−0.005	0.007
SA → BPD features	−0.093	0.048	−0.187	0.004
Model 4B - Three types of childhood abuse, two simple mediators (resilience and self-esteem) and one three-path mediator (self-esteem to resilience)				
EA → resilience → BPD features	0.013^*^	0.006	0.001	0.025
EA → self-esteem → BPD features	0.036^**^	0.008	0.024	0.053
EA → self-esteem → resilience → BPD features	0.033^**^	0.006	0.023	0.046
EA → BPD features	0.371^**^	0.044	0.289	0.458
PA → resilience → BPD features	0.001	0.008	−0.014	0.017
PA → self-esteem → BPD features	− 0.001	0.007	− 0.014	0.012
PA → self-esteem → resilience → BPD features	−0.001	0.006	−0.013	0.011
PA → BPD features	−0.041	0.057	−0.152	0.071
SA → resilience → BPD features	0.003	0.008	−0.013	0.019
SA → self-esteem → BPD features	0.000	0.005	−0.012	0.010
SA → self-esteem → resilience → BPD features	0.000	0.005	−0.010	0.009
SA → BPD features	−0.093	0.048	−0.187	0.004
Model 4C - Three types of childhood abuse, two simple mediators (resilience and self-esteem)				
EA → resilience → BPD features	0.061^**^	0.012	0.041	0.086
EA → self-esteem → BPD features	0.045^**^	0.010	0.029	0.067
EA → BPD features	0.380^**^	0.046	0.295	0.472
PA → resilience → BPD features	−0.014	0.015	− 0.047	0.013
PA → self-esteem → BPD features	− 0.009	0.010	− 0.030	0.010
PA → BPD features	− 0.045	0.058	− 0.160	0.070
SA → resilience → BPD features	0.004	0.011	−0.017	0.026
SA → self-esteem → BPD features	0.000	0.007	−0.014	0.014
SA → BPD features	−0.094	0.049	−0.190	0.005

*EA* emotional abuse, *PA* physical abuse, *SA* sexual abuse, *SE* Standard Error, lower lower bound of 95% confidence interval, upper upper bound of 95% confidence interval. ^**^P < 0.001, ^*^P < 0.05

## Discussion

The results of Model A, Model B and Model C were similar, indicating that no matter what the relationship between self-esteem and resilience is, self-esteem and resilience mediated the links between childhood abuse and BPD features, and emotional abuse is uniquely associated with BPD features. First, we found that after controlling for the overlap between the three types of childhood abuse, only emotional abuse was uniquely associated with BPD features, which may reflect the special effects of emotional abuse on the development of BPD features. This result was consistent with previous findings [12–14]. For example, Kuo et al. [14] found that only emotional abuse (and not other forms of abuse) was uniquely associated with the severity of BPD features and that difficulties with emotion regulation mediated the relationship when other forms of abuse were controlled.

It is logical that in an emotionally abusive rearing environment, individuals form a set of negative perceptions about themselves and fail to develop the ability that responding to changing environmental circumstances flexibly, as the experience of emotional abuse could directly convey a negative self-image that one is worthless, flawed, unloved or unwanted [53]. It is unsurprising that emotional abuse subsequently affects the healthy development of personality and leads to BPD features. Therefore, we could infer from our results that emotional abuse has a more pronounced impact on personality health development than physical abuse or sexual abuse, which is consistent with previous research showing that emotional abuse is central to childhood abuse and may be more harmful than other forms of abuse [32, 53–55]. Compared to other forms of abuse, emotional abuse was found to result in a higher risk of developing mental disorders and psychological symptoms [32, 55].

Second, the mediation analysis showed that the association between emotional abuse and BPD features was partially mediated by resilience, self-esteem. Emotional abuse had not only a direct impact on BPD features but also an indirect impact via resilience and self-esteem. This finding is in line with numerous previous studies that self-esteem and resilience could mediate the relationship between childhood emotional abuse and mental health problems, such as emotional and behavioral problems, adulthood psychopathology, and psychological symptoms [32–34]. Base on the emotional flexibility theory, high resilience can help people flexibly change their affective and physiological responses, as reflected in a low level of emotional variability [30]. On the contrary, a high level of emotional variability is a hallmark of BPD symptoms [56]. Self-esteem could serve as a positive functioning dimension that helps adolescents manage, regulate, and minimize their psychological distress to build higher levels of subjective happiness, which is beneficial for their mental health [57]. The diathesis-stress model suggests that the more vulnerable the person, the less environmental stress was required to develop BPD features [58]. The results of our study confirm this model and prove that low resilience and self-esteem would promote the development of BPD features.

### Limitations

Although our study has a number of strengths, including the large sample size, the adoption of a random sampling approach and the separate and simultaneous testing of three types of childhood abuse, our study has several limitations. First, given that the cross-sectional design of our study can’t provide time-ordering of resilience, self-esteem, and BPD features, there may be other models [59]. Future prospective researches are required to explore the relationship between childhood abuse, resilience, self-esteem, and BPD features. Second, childhood abuse, resilience, self-esteem, and BPD features were assessed by self-report; thus, recall bias was inevitable. Third, the sociodemographic variables such as age, gender, area of family residence, grade and academic disciplines were not included in these models, in other words, we did not control for the sociodemographic variables. Fourth, only four universities in one city were included in this study, so the conclusions cannot be extended to all college students in China.

## Conclusions

Firstly, the findings of our research provide valuable insight into the relationship between childhood abuse, self-esteem, resilience, and BPD features among Chinese college students. Secondly, our study provides novel evidence that emotional abuse was uniquely associated with BPD features. Thirdly, our study provides novel evidence that self-esteem and resilience are important protective factors between childhood abuse and BPD features. To our knowledge, our study is the first study to investigate the mechanism of the relationship among childhood abuse, resilience, self-esteem and BPD features, thus our research fills the gap in this field and expands the relationship between childhood abuse and BPD features. Furthermore, our study could provide school staff and mental health professionals with a number of meaningful guidance. For example, given our finding that self-esteem and resilience may be important intervention factors for controlling the development of BPD features in the context of childhood abuse, school staff and mental health professionals could consider improving an individual’s resilience and self-esteem to control the development of BPD features. Likewise, our finding that emotional abuse uniquely relates to BPD features suggests great importance should be attached to emotionally abused victims.

## Supplementary Information

**Additional file 1.**

**Additional file 2.**

**Additional file 3.**

**Additional file 4.**

**Additional file 5.**

**Additional file 6.**

## Data Availability

All data and materials related to the study are available from the corresponding author upon reasonable request.

## Abbreviations

BPD: Borderline personality disorder
SEM: Structural equation modeling
EA: Emotional abuse
PA: Physical abuse
SA: Sexual abuse
CTQ-SF: Childhood Trauma Questionnaire–Short Form
MSI-BPD: Mclean Screening Instrument for Borderline Personality Disorder
CD-RISC: Connor-Davidson Resilience Scale
RSES: Rosenberg Self-Esteem Scale
