# Supplementary material for: Childhood abuse and borderline personality disorder features in Chinese undergraduates: the role of self-esteem and resilience
Source: BMC Psychiatry. 2021 Jul 1;21:326. doi: 10.1186/s12888-021-03332-w (PMC8252225; doi:10.1186/s12888-021-03332-w)
Supplement: Supplementary file 1 — Additional file 1. [file 12888_2021_3332_MOESM1_ESM.docx]

**Model 1A**


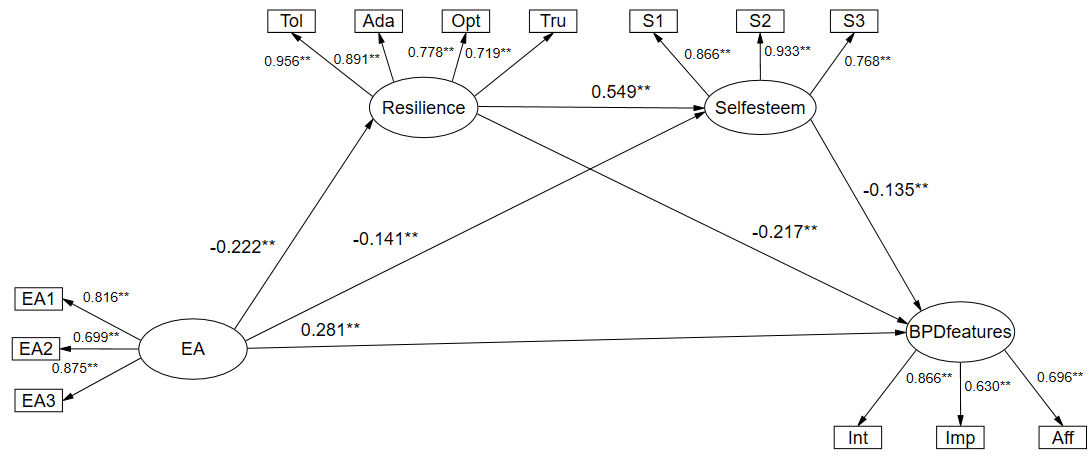


**Model 1B**


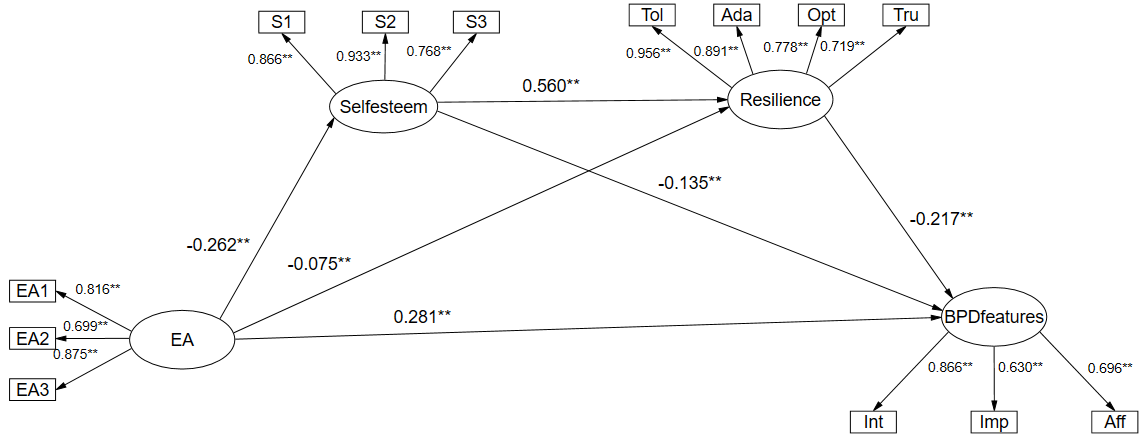


**Model 1C**


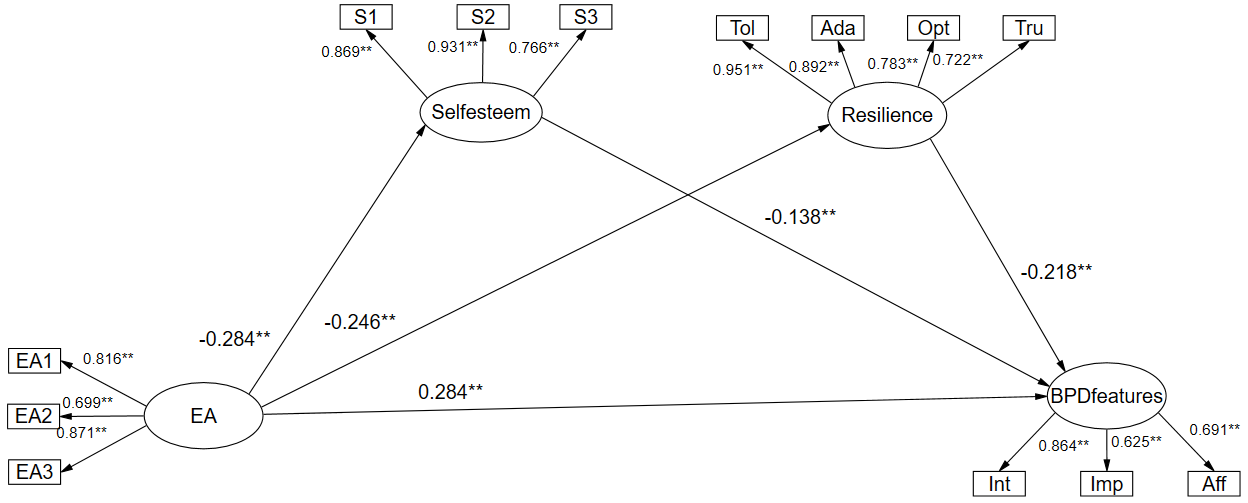


**Additional file 1** The mediating effects of resilience and self-esteem when emotional abuse was examined individually (Model 1).

Note. This figure depicts standardized regression weights. The first model is (1a), the second model is (1b) and the third model is (1c). EA emotional abuse. Model fit indices for Model 1A and 1B: CFI=0.999, TLI=0.999, RMSEA=0.009, χ^2^=79.011, df=59, χ^2^/df=1.339; Model fit indices for Model 1C: CFI=0.999, TLI=0.999, RMSEA=0.009, χ^2^=80.487, df=60, χ^2^/df=1.342. ^**^*P* < 0.001, ^*^*P* < 0.05.
