# Supplementary material for: Childhood abuse and borderline personality disorder features in Chinese undergraduates: the role of self-esteem and resilience
Source: BMC Psychiatry. 2021 Jul 1;21:326. doi: 10.1186/s12888-021-03332-w (PMC8252225; doi:10.1186/s12888-021-03332-w)
Supplement: Supplementary file 2 — Additional file 2. [file 12888_2021_3332_MOESM2_ESM.docx]

**Model 2A**


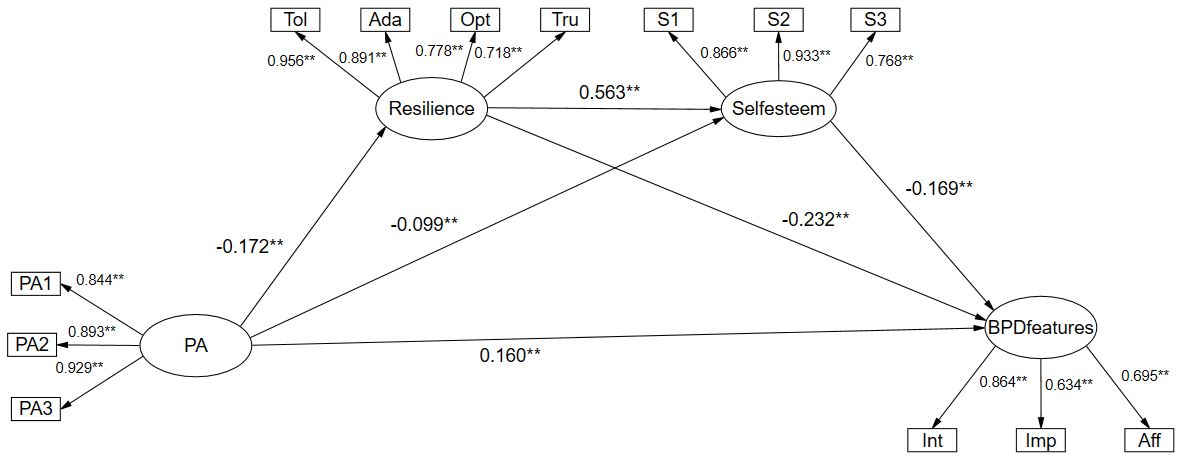


**Model 2B**


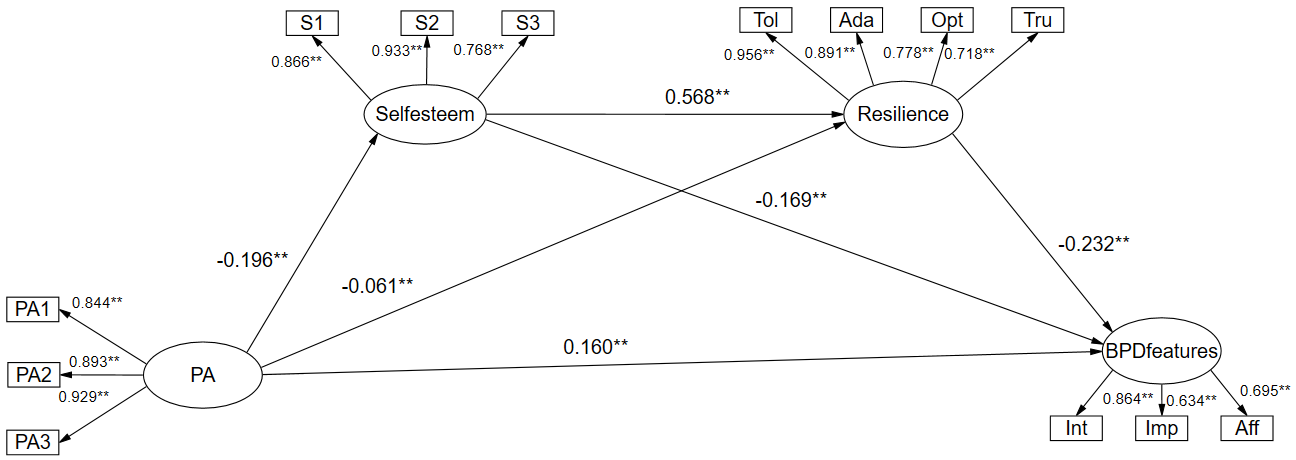


**Model 2C**


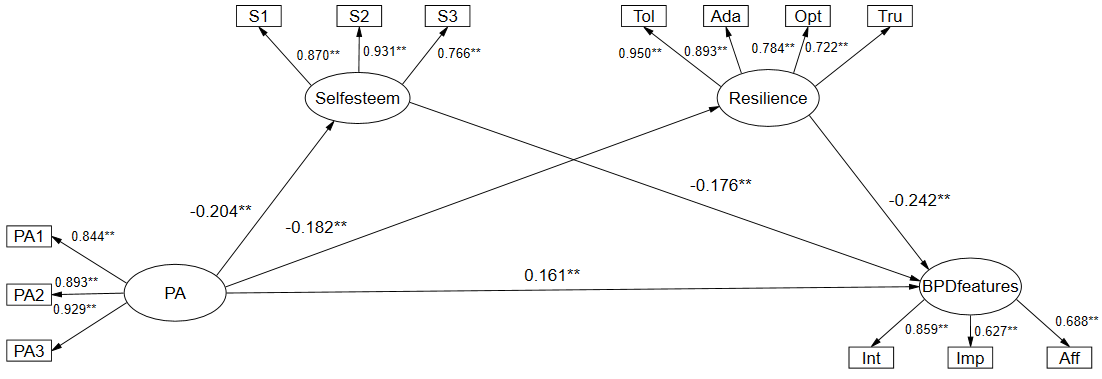


**Additional file 2** The mediating effects of resilience and self-esteem when physical abuse was examined individually (Model 2).

Note. This figure depicts standardized regression weights. The first model is (2a), the second model is (2b) and the third model is (2c). PA physical abuse. Model fit indices for Model 2A and 2B: CFI=0.999, TLI=0.999, RMSEA=0.010, χ^2^=80.581, df=59, χ^2^/df=1.366; Model fit indices for Model 2C: CFI=0.999, TLI=0.999, RMSEA=0.010, χ^2^=82.091, df=60, χ^2^/df=1.368. ^**^*P* < 0.001, ^*^*P* < 0.05.
