# Supplementary material for: Childhood abuse and borderline personality disorder features in Chinese undergraduates: the role of self-esteem and resilience
Source: BMC Psychiatry. 2021 Jul 1;21:326. doi: 10.1186/s12888-021-03332-w (PMC8252225; doi:10.1186/s12888-021-03332-w)
Supplement: Supplementary file 3 — Additional file 3. [file 12888_2021_3332_MOESM3_ESM.docx]

**Model 3A**


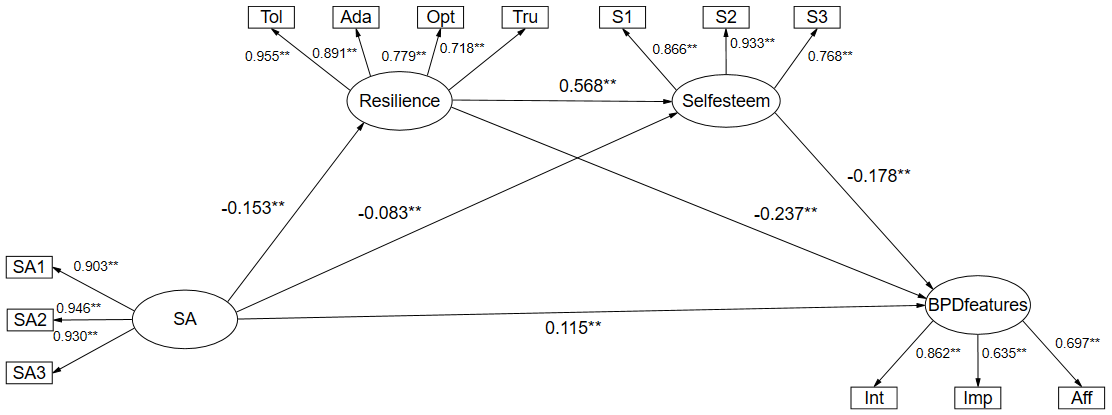


**Model 3B**


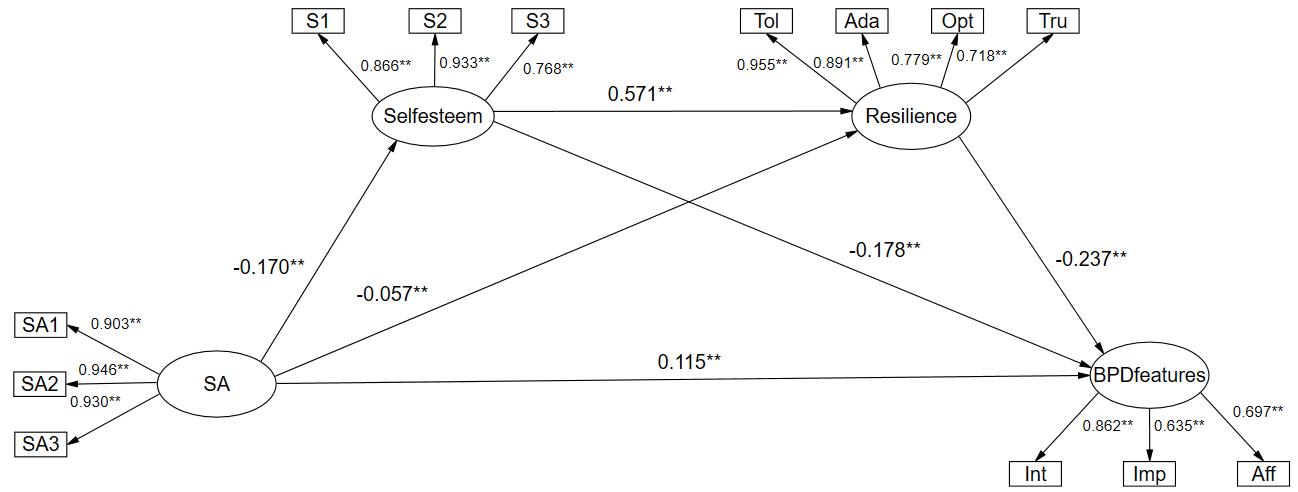


**Model 3C**


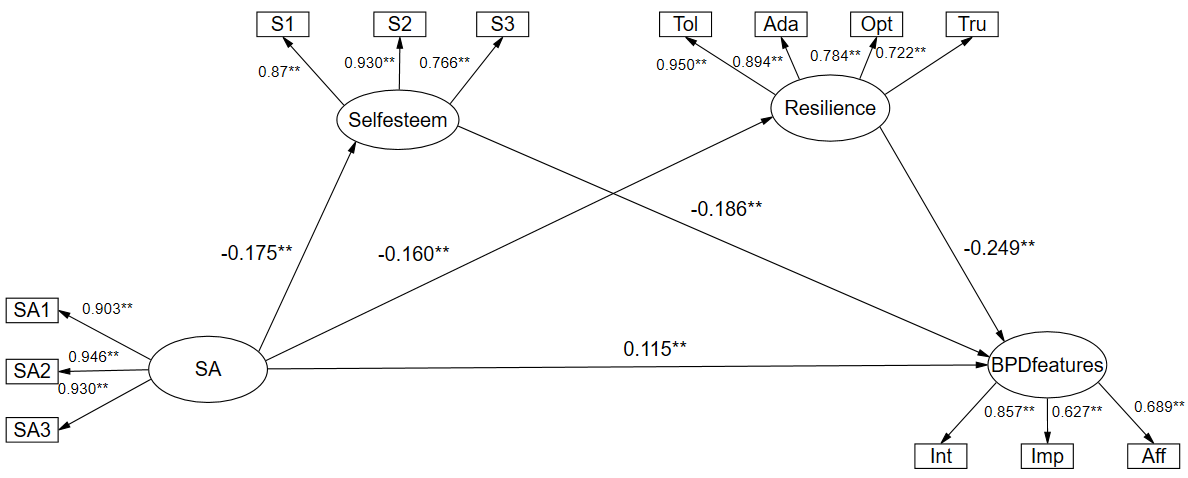


**Additional file 3** The mediating effects of resilience and self-esteem when sexual abuse was examined individually (Model 3).

Note. This figure depicts standardized regression weights. The first model is (3a), the second model is (3b) and the third model is (3c). SA sexual abuse. Model fit indices for Model 3A and 3B: CFI=0.999, TLI=0.999, RMSEA=0.011, χ^2^=89.614, df=59, χ^2^/df=1.519; Model fit indices for Model 3C: CFI=0.999, TLI=0.999, RMSEA=0.011, χ^2^=91.118, df=60, χ^2^/df=1.519. ^**^*P* < 0.001, ^*^*P* < 0.05.
