# Supplementary material for: Childhood abuse and borderline personality disorder features in Chinese undergraduates: the role of self-esteem and resilience
Source: BMC Psychiatry. 2021 Jul 1;21:326. doi: 10.1186/s12888-021-03332-w (PMC8252225; doi:10.1186/s12888-021-03332-w)
Supplement: Supplementary file 4 — Additional file 4. [file 12888_2021_3332_MOESM4_ESM.docx]

**Additional file 4** Indirect and direct effects of childhood abuse on BPD features – emotional abuse examined individually (Model 1)

| Model pathway | Estimate | SE | lower | upper |
| --- | --- | --- | --- | --- |
| Model 1A - Emotional abuse, two simple mediators (resilience and self-esteem) and one three-path mediator (resilience to self-esteem) | | | | |
| EA → resilience → BPD features | 0.048^**^ | 0.007 | 0.035 | 0.063 |
| EA → self-esteem → BPD features | 0.019^**^ | 0.004 | 0.012 | 0.028 |
| EA → resilience → self-esteem → BPD features | 0.016^**^ | 0.004 | 0.010 | 0.024 |
| EA →BPD features | 0.281^**^ | 0.026 | 0.230 | 0.332 |
| Model 1B - Emotional abuse, two simple mediators (resilience and self-esteem) and one three-path mediator (self-esteem to resilience) | | | | |
| EA → resilience → BPD features | 0.016^**^ | 0.005 | 0.006 | 0.027 |
| EA → self-esteem → BPD features | 0.036^**^ | 0.006 | 0.024 | 0.049 |
| EA →self-esteem → resilience → BPD features | 0.032^**^ | 0.005 | 0.023 | 0.042 |
| EA → BPD features | 0.281^**^ | 0.026 | 0.230 | 0.332 |
| Model 1C - Emotional abuse, two simple mediators (resilience and self-esteem) | | | | |
| EA → resilience → BPD features | 0.054^**^ | 0.008 | 0.039 | 0.070 |
| EA → self-esteem → BPD features | 0.039^**^ | 0.007 | 0.027 | 0.053 |
| EA → BPD features | 0.284^**^ | 0.026 | 0.233 | 0.336 |

Note. EA emotional abuse, lower lower bound of 95% confidence interval, upper upper bound of 95% confidence interval. ^**^*P* < 0.001, ^*^*P* < 0.05.
