# Supplementary material for: Childhood abuse and borderline personality disorder features in Chinese undergraduates: the role of self-esteem and resilience
Source: BMC Psychiatry. 2021 Jul 1;21:326. doi: 10.1186/s12888-021-03332-w (PMC8252225; doi:10.1186/s12888-021-03332-w)
Supplement: Supplementary file 5 — Additional file 5. [file 12888_2021_3332_MOESM5_ESM.docx]

**Additional file 5** Indirect and direct effects of childhood abuse on BPD features – physical abuse examined individually (Model 2)

| Model pathway | Estimate | SE | lower | upper |
| --- | --- | --- | --- | --- |
| Model 2A - Physical abuse, two simple mediators (resilience and self-esteem) and one three-path mediator (resilience to self-esteem) | | | | |
| PA → resilience → BPD features | 0.040^**^ | 0.007 | 0.028 | 0.054 |
| PA → self-esteem → BPD features | 0.017^**^ | 0.004 | 0.009 | 0.025 |
| PA → resilience → self-esteem → BPD features | 0.016^**^ | 0.003 | 0.011 | 0.024 |
| PA →BPD features | 0.160^**^ | 0.027 | 0.110 | 0.215 |
| Model 2B - Physical abuse, two simple mediators (resilience and self-esteem) and one three-path mediator (self-esteem to resilience) | | | | |
| PA → resilience → BPD features | 0.014^*^ | 0.006 | 0.004 | 0.027 |
| PA → self-esteem → BPD features | 0.033^**^ | 0.005 | 0.024 | 0.044 |
| PA →self-esteem → resilience → BPD features | 0.026^**^ | 0.004 | 0.019 | 0.034 |
| PA → BPD features | 0.160^**^ | 0.027 | 0.110 | 0.215 |
| Model 2C - Physical abuse, two simple mediators (resilience and self-esteem) | | | | |
| PA → resilience → BPD features | 0.044^**^ | 0.007 | 0.031 | 0.059 |
| PA → self-esteem → BPD features | 0.036^**^ | 0.005 | 0.027 | 0.047 |
| PA → BPD features | 0.161^**^ | 0.027 | 0.110 | 0.217 |

Note. PA physical abuse, lower lower bound of 95% confidence interval, upper upper bound of 95% confidence interval. ^**^*P* < 0.001, ^*^*P* < 0.05.
