# Supplementary material for: Childhood abuse and borderline personality disorder features in Chinese undergraduates: the role of self-esteem and resilience
Source: BMC Psychiatry. 2021 Jul 1;21:326. doi: 10.1186/s12888-021-03332-w (PMC8252225; doi:10.1186/s12888-021-03332-w)
Supplement: Supplementary file 6 — Additional file 6. [file 12888_2021_3332_MOESM6_ESM.docx]

**Additional file 6** Indirect and direct effects of childhood abuse on BPD features – sexual abuse examined individually (Model 3)

| Model pathway | Estimate | SE | lower | upper |
| --- | --- | --- | --- | --- |
| Model 3A - Sexual abuse, two simple mediators (resilience and self-esteem) and one three-path mediator (resilience to self-esteem) | | | | |
| SA → resilience → BPD features | 0.036^**^ | 0.007 | 0.024 | 0.050 |
| SA → self-esteem → BPD features | 0.015^**^ | 0.004 | 0.007 | 0.024 |
| SA → resilience → self-esteem → BPD features | 0.016^**^ | 0.003 | 0.010 | 0.023 |
| SA →BPD features | 0.115^**^ | 0.028 | 0.061 | 0.171 |
| Model 3B - Sexual abuse, two simple mediators (resilience and self-esteem) and one three-path mediator (self-esteem to resilience) | | | | |
| SA → resilience → BPD features | 0.013^*^ | 0.006 | 0.002 | 0.027 |
| SA → self-esteem → BPD features | 0.030^**^ | 0.005 | 0.022 | 0.040 |
| SA →self-esteem → resilience → BPD features | 0.023^**^ | 0.004 | 0.016 | 0.031 |
| SA → BPD features | 0.115^**^ | 0.028 | 0.061 | 0.171 |
| Model 3C - Sexual abuse, two simple mediators (resilience and self-esteem) | | | | |
| SA → resilience → BPD features | 0.040^**^ | 0.007 | 0.027 | 0.054 |
| SA → self-esteem → BPD features | 0.033^**^ | 0.005 | 0.024 | 0.043 |
| SA → BPD features | 0.115^**^ | 0.029 | 0.060 | 0.172 |

Note. SA sexual abuse, lower lower bound of 95% confidence interval, upper upper bound of 95% confidence interval. ^**^*P* < 0.001, ^*^*P* < 0.05.
